# Supplementary material for: End Sequence Analysis Toolkit (ESAT) expands the extractable information from single-cell RNA-seq data
Source: Genome Res. 2016 Oct;26(10):1397–410. doi: 10.1101/gr.207902.116 (PMC5052061; doi:10.1101/gr.207902.116)
Supplement: Supplemental Material [file supp_gr.207902.116_Supp_Materials.tar.gz › ESAT-esat_v0.1/doc/html/interfacebroad_1_1core_1_1annotation_1_1_two_subject_annotation.html]

ESAT: broad.core.annotation.TwoSubjectAnnotation Interface Reference


ESAT


- Main Page
- Related Pages
- Packages
- Classes
- Files

- Class List
- Class Index
- Class Hierarchy
- Class Members

All Classes Namespaces Files Functions Variables Enumerator Pages

- broad
- core
- annotation
- TwoSubjectAnnotation

Public Member Functions |
List of all members

broad.core.annotation.TwoSubjectAnnotation Interface Reference

Inheritance diagram for broad.core.annotation.TwoSubjectAnnotation:

[legend]

|  |  |
| --- | --- |
| Public Member Functions | |
| int | getAStart () |
|  | |
| int | getBStart () |
|  | |
| int | getAEnd () |
|  | |
| int | getBEnd () |
|  | |
| String | getAName () |
|  | |
| String | getBName () |
|  | |
| LightweightGenomicAnnotation | getA () |
|  | |
| LightweightGenomicAnnotation | getB () |
|  | |
| boolean | isDirect () |
|  | |

## Member Function Documentation

|  |  |  |  |  |
| --- | --- | --- | --- | --- |
| LightweightGenomicAnnotation broad.core.annotation.TwoSubjectAnnotation.getA | ( |  | ) |  |

Implemented in broad.core.annotation.BasicTwoSubjectAnnotation.

|  |  |  |  |  |
| --- | --- | --- | --- | --- |
| int broad.core.annotation.TwoSubjectAnnotation.getAEnd | ( |  | ) |  |

Implemented in broad.core.annotation.BasicTwoSubjectAnnotation.

|  |  |  |  |  |
| --- | --- | --- | --- | --- |
| String broad.core.annotation.TwoSubjectAnnotation.getAName | ( |  | ) |  |

Implemented in broad.core.annotation.BasicTwoSubjectAnnotation.

|  |  |  |  |  |
| --- | --- | --- | --- | --- |
| int broad.core.annotation.TwoSubjectAnnotation.getAStart | ( |  | ) |  |

Implemented in broad.core.annotation.BasicTwoSubjectAnnotation.

|  |  |  |  |  |
| --- | --- | --- | --- | --- |
| LightweightGenomicAnnotation broad.core.annotation.TwoSubjectAnnotation.getB | ( |  | ) |  |

Implemented in broad.core.annotation.BasicTwoSubjectAnnotation.

|  |  |  |  |  |
| --- | --- | --- | --- | --- |
| int broad.core.annotation.TwoSubjectAnnotation.getBEnd | ( |  | ) |  |

Implemented in broad.core.annotation.BasicTwoSubjectAnnotation.

|  |  |  |  |  |
| --- | --- | --- | --- | --- |
| String broad.core.annotation.TwoSubjectAnnotation.getBName | ( |  | ) |  |

Implemented in broad.core.annotation.BasicTwoSubjectAnnotation.

|  |  |  |  |  |
| --- | --- | --- | --- | --- |
| int broad.core.annotation.TwoSubjectAnnotation.getBStart | ( |  | ) |  |

Implemented in broad.core.annotation.BasicTwoSubjectAnnotation.

|  |  |  |  |  |
| --- | --- | --- | --- | --- |
| boolean broad.core.annotation.TwoSubjectAnnotation.isDirect | ( |  | ) |  |

Implemented in broad.core.annotation.BasicTwoSubjectAnnotation.

---

The documentation for this interface was generated from the following file:

- java/broad/core/annotation/TwoSubjectAnnotation.java


---

Generated on Fri Aug 15 2014 15:10:51 for ESAT by  

 1.8.7
